# Increased Population Prevalence of Low Pertussis Toxin Antibody Levels in Young Children Preceding a Record Pertussis Epidemic in Australia

**DOI:** 10.1371/journal.pone.0035874

**Published:** 2012-04-27

**Authors:** Patricia Campbell, Peter McIntyre, Helen Quinn, Linda Hueston, Gwendolyn L. Gilbert, Jodie McVernon

**Affiliations:** 1 Vaccine & Immunisation Research Group, Murdoch Childrens Research Institute and Melbourne School of Population Health, The University of Melbourne, Parkville, Victoria, Australia; 2 National Centre for Immunisation Research & Surveillance of Vaccine Preventable Diseases, The Children's Hospital at Westmead, Sydney, New South Wales, Australia; 3 Centre for Infectious Diseases and Microbiology - Public Health, Institute for Clinical Pathology and Medical Research, Westmead Hospital, Sydney, New South Wales, Australia; Instituto Butantan, Brazil

## Abstract

**Background:**

Cross-sectional serosurveys using IgG antibody to pertussis toxin (IgG-PT) are increasingly being used to estimate trends in recent infection independent of reporting biases.

**Methods/Principal Findings:**

We compared the age-specific seroprevalence of various levels of IgG-PT in cross-sectional surveys using systematic collections of residual sera from Australian diagnostic laboratories in 1997/8, 2002 and 2007 with reference to both changes in the pertussis vaccine schedule and the epidemic cycle, as measured by disease notifications. A progressive decline in high-level (≥62.5 EU/ml) IgG-PT prevalence from 19% (95% CI 16–22%) in 1997/98 to 12% (95% CI 11–14%) in 2002 and 5% (95% CI 4–6%) in 2007 was consistent with patterns of pertussis notifications in the year prior to each collection. Concomitantly, the overall prevalence of undetectable (<5 EU/ml) levels increased from 17% (95% CI 14–20%) in 1997/98 to 38% (95% CI 36–40%) in 2007 but among children aged 1–4 years, from 25% (95% CI 17–34%) in 1997/98 to 62% (95% CI 56–68%) in 2007. This change followed withdrawal of the 18-month booster dose in 2003 and preceded record pertussis notifications from 2008 onwards.

**Conclusions/Significance:**

Population seroprevalence of high levels of IgG-PT is accepted as a reliable indicator of pertussis disease activity over time within and between countries with varying diagnostic practices, especially in unimmunised age groups. Our novel findings suggest that increased prevalence of undetectable IgG-PT is an indicator of waning immunity useful for population level monitoring following introduction of acellular vaccines and/or schedule changes.

## Introduction

Pertussis, also known as whooping cough, is a highly contagious respiratory disease caused by infection with the bacterium *Bordetella pertussis*. The most severe symptoms occur in childhood, and infants too young to be vaccinated account for most deaths [1]. In 2008, there were an estimated 16 million cases of pertussis worldwide, resulting in the deaths of 195,000 children, mostly in developing countries [2]. Many countries with well established, high coverage vaccination programs are experiencing increasing notifications, and a shift in disease burden from children to adults [3].

**Figure 1:**
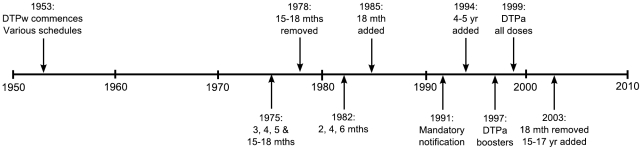
Timeline of major vaccination and reporting changes in Australia from 1953 to present. The timeline covers the commencement of vaccination in 1953 through various schedule changes, the introduction of mandatory notification and changes to the vaccine type used in Australia.

Universal pertussis vaccination, using a combined diphtheria, tetanus and whole cell pertussis vaccine (DTPw) commenced in Australia in 1953 [4]. Since then, a number of changes have been made to the timing of scheduled doses. Major schedule changes in the last twenty years have been the addition of a pre-school booster in 1994 to reduce levels of transmission in school aged children [5], and the replacement of the 18 month booster with an adolescent booster in 2003, to address increasing notifications in adolescents [6]. Further, in the late 1990s, acellular vaccines (DTPa) replaced the Australian-manufactured whole cell vaccine [7]. Almost all acellular vaccines used in Australia have been GlaxoSmithKline three component formulations, in various age appropriate concentrations and combinations. The timeline of changes, which have created many cohorts with different pertussis vaccination experiences, is provided in Figure 1. Since 2003, the Australian National Immunisation Program (NIP) pertussis schedule has consisted of a primary course at 2, 4, and 6 months, with boosters at 4 years and 15–17 years, all with acellular vaccines [7].

Vaccination coverage in Australia increased through the 1990s and has been relatively stable at high levels since 2000. Reported coverage in 2007 showed 92% of children nationally complete the pertussis primary vaccination course by 12 months, rising to 95% by two years of age [8]. Coverage estimates at the State level are generally similar to National figures, although there is variation at the finer geographic level of Australian Bureau of Statistics statistical subdivision (SSD) [8]. In 2007, at least 85% of 12 month old children in each SSD had completed the primary vaccination course, with the exception of a small number of under-vaccinated jurisdictions [8]. In 2007, around 89% of children received the pertussis pre-school booster by six years of age [8].

In Australia, the use of pertussis notification rates to analyse long term disease trends is confounded by changes in disease awareness, increasing use of diagnostic testing and availability of sensitive PCR diagnostic methods compared to earlier less sensitive culture methods [9]. National notifications remained in a relatively stable 3–4 year epidemic cycle from 1995 to 2005, obscuring the underlying regional variability in cycle length between states with different population densities [10]. From 2008 to 2011, however, both national and state notification rates persisted at epidemic levels [11]. Although possibly influenced by the increased use of PCR for diagnosis, the correlation with hospitalisations indicates a genuine increase in the number of cases [12]. Notably, increased notifications in children aged less than 10 years old were observed during the 2008–2011 epidemic, although these ages were previously thought to be well protected by vaccination.

Cross-sectional population serological surveys for pertussis measure serum IgG antibodies to pertussis toxin (IgG-PT). An IgG-PT level of 125 ELISA U/ml (EU/ml) or more is a highly sensitive and specific marker of recent or active infection with *B. pertussis* [13]. High sensitivity and specificity are major advantages of serosurveillance over traditional forms of pertussis surveillance, which are hampered by variable symptoms, diagnostic algorithms and the dependence of diagnostic test sensitivity on age, previous infections, vaccination status and time elapsed since the onset of infection [1]. Serosurveillance can better estimate the population level of infection as mild and asymptomatic cases can be detected, allowing objective comparisons over time and between countries using standardised assays.

This study analysed the cross-sectional population distribution of IgG-PT levels during a period of low pertussis disease activity in Australia and compared the results with two previous serosurveys undertaken at different stages of the pertussis epidemic cycle. The aim was to evaluate trends over time and explore links between vaccination, the epidemic cycle and the cross-sectional serological profile in age groups eligible and not eligible for routine immunisation.

## Methods

### Ethics statement

Approval for the serosurvey was obtained from the Sydney West Area Health Service Human Research Ethics Committee. Informed consent was not obtained as the data were analysed anonymously. In accordance with the provisions of the National Health and Medical Research Council's National Statement on Ethical Conduct in Human Research, individual consent was not required for use of these specimens, given the granting of institutional approval by the Sydney West Area Health Service Human Research Ethics Committee.

### Study population and design

This study reports on findings from a serosurvey undertaken in 2007 for the purposes of evaluating immunity in Australia to a range of vaccine preventable diseases, including pertussis. The study design and laboratory methods are the same as those used for the 1997/98 and 2002 serosurveys [4], [7]. Briefly, the sera used were randomly selected from a bank of approximately 7200 sera collected under Australia's national serosurveillance program. These were residual samples from specimens submitted for diagnostic testing to a nationally representative group of laboratories and would otherwise have been discarded. Sera from subjects who were immunosuppressed, had received multiple or recent (within three months) blood transfusions, or were known to be infected with HIV were excluded. Residual samples are often used for pertussis serosurveillance [14]–[16], and this type of study design has been shown to achieve similar results to population-based cluster sampling for the measurement of immunity to measles and mumps [17]. Sera were identified by sex, age, address postcode (of the subject, if available, or the supplying laboratory) and a unique identifier. Clinical information and pertussis vaccination status were not collected.

### Assay methods

A total of 3224 sera were randomly selected from the available samples to fulfil calculated sample size requirements in each age group, commencing at one year of age. Measurement of IgG-PT levels was undertaken using an established ELISA method adapted from Giammanco et al. [18] and described by Quinn et al. [7]. The same laboratory method was used for the 1997/98, 2002 and 2007 studies, with the 1997/98 analysis conducted at the European Sero-Epidemiology Network (ESEN) reference laboratory at the University of Palermo, Italy and the 2002 and 2007 analyses performed at the Centre for Infectious Diseases and Microbiology (CIDM), Sydney, Australia. The method used at the CIDM laboratory was validated against a panel of sera from the ESEN reference laboratory.

The minimum level of detection of the assay was estimated to be 2 EU/ml. Antibody levels were divided into four categories, suggestive of pertussis infection or vaccination, with immune response, within the following time periods [15]: <5 EU/ml (undetectable), 5–<62.5 EU/ml (mid-range: more than one year previously), 62.5–<125 EU/ml (high: within 12 months) and ≥125 EU/ml (very high: within 6 months).

### Pertussis surveillance data

In Australia, notification of both confirmed and probable pertussis cases to the National Notifiable Diseases Surveillance System (NNDSS) is mandatory [19]. According to the NNDSS definition, confirmed cases require either laboratory definitive evidence (“isolation of *Bordetella pertussis* or detection of *B. pertussis* by nucleic acid testing”), or laboratory suggestive evidence (“seroconversion or significant increase in antibody level or fourfold or greater rise in titre to *B. pertussis* in the absence of recent pertussis vaccination; or single high IgA titre to whole cells; or detection of *B. pertussis* antigen by immunofluorescence assay”) combined with clinical evidence (“a coughing illness lasting two or more weeks; or paroxysms of coughing or inspiratory whoop or post-tussive vomiting”), or clinical evidence combined with an epidemiological link to a confirmed case [19]. Probable cases are notified on the basis of clinical evidence only [19]; however the strong reliance on direct notification from laboratories in Australia and poor notification from clinicians [20] suggest that the great majority of cases are now notified based on positive PCR or serology. Annual pertussis notifications for the period 1991–2011 were obtained from published NNDSS data [11]. Incidence rates per 100,000 population were calculated using these published notification numbers, and Australian Bureau of Statistics mid year population estimates [21].

### Statistical methods

Age specific prevalence of IgG-PT levels in each category was calculated, along with Clopper-Pearson confidence intervals. Bonferroni corrections were applied to ensure a family confidence level of 95% for comparisons across all years, equivalent to individual confidence levels of 98.3%. Differences between groups were considered statistically significant at the 5% level when these confidence intervals did not overlap. Analysis was undertaken in Microsoft Office Excel 2003 (Microsoft Corporation) and STATA, version 11.2 (StataCorp).

## Results

### Epidemiologic context

**Figure 2:**
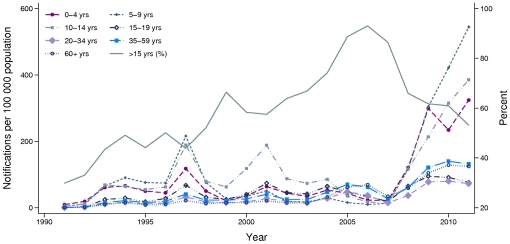
Age specific pertussis notification rates per 100,000 population for the period 1991 to 2011. In addition to the age specific notification rates per 100,000 population, this graph includes a trend line showing the percentage of notifications occurring in over 15 year olds over the same period.

Age specific notification rates per 100,000 population for 1991 to 2011 are shown in Figure 2, along with a trend line detailing the percentage of cases in ages ≥15 years. Epidemics occur approximately every three to four years against a background of endemic disease. Serosurvey collections occurred at different stages of the pertussis cycle: 1997/98 during and immediately following an epidemic year; 2002 during a period of moderate endemic activity; and 2007 at a trough in observed disease, prior to a large epidemic sustained over the period 2008–2011. Yearly notifications for the period 2008–2011 totalled 14292, 29799, 34793 and 38131 respectively.

Throughout the 1990s, the percentage of cases in <15 year olds gradually decreased, and from 1998–2011 most cases have occurred in those aged ≥15 years and over. This reached a maximum in 2006, when more than 90% of cases were notified in the ≥15 years age group. During the 2008–2011 epidemic, however, the percentage of notifications in these older ages decreased. In particular, whilst only 15% of notifications occurred in <10 year olds for the period 1999–2004, this rose to over 30% in 2011.

The highest notification rates during the 2008–2011 epidemic occurred in the 0–4, 5–9 and 10–14 years age groups. In 2011, notification rates in these three age groups (324, 385 and 544 cases per 100,000 population respectively) were more than double those in 1997, their maximums before this epidemic. Notification rates in the 35–59 and ≥60 years age groups also rose sharply in 2008 and by 2010 the rates in these age groups (140 and 128 cases per 100,000 population respectively) were also double their previous maximums. The lowest rates during the 2008–2011 epidemic were experienced by the 15–19 and 20–34 years age groups.

### Overall trends in the population distribution of IgG-PT levels

**Figure 3:**
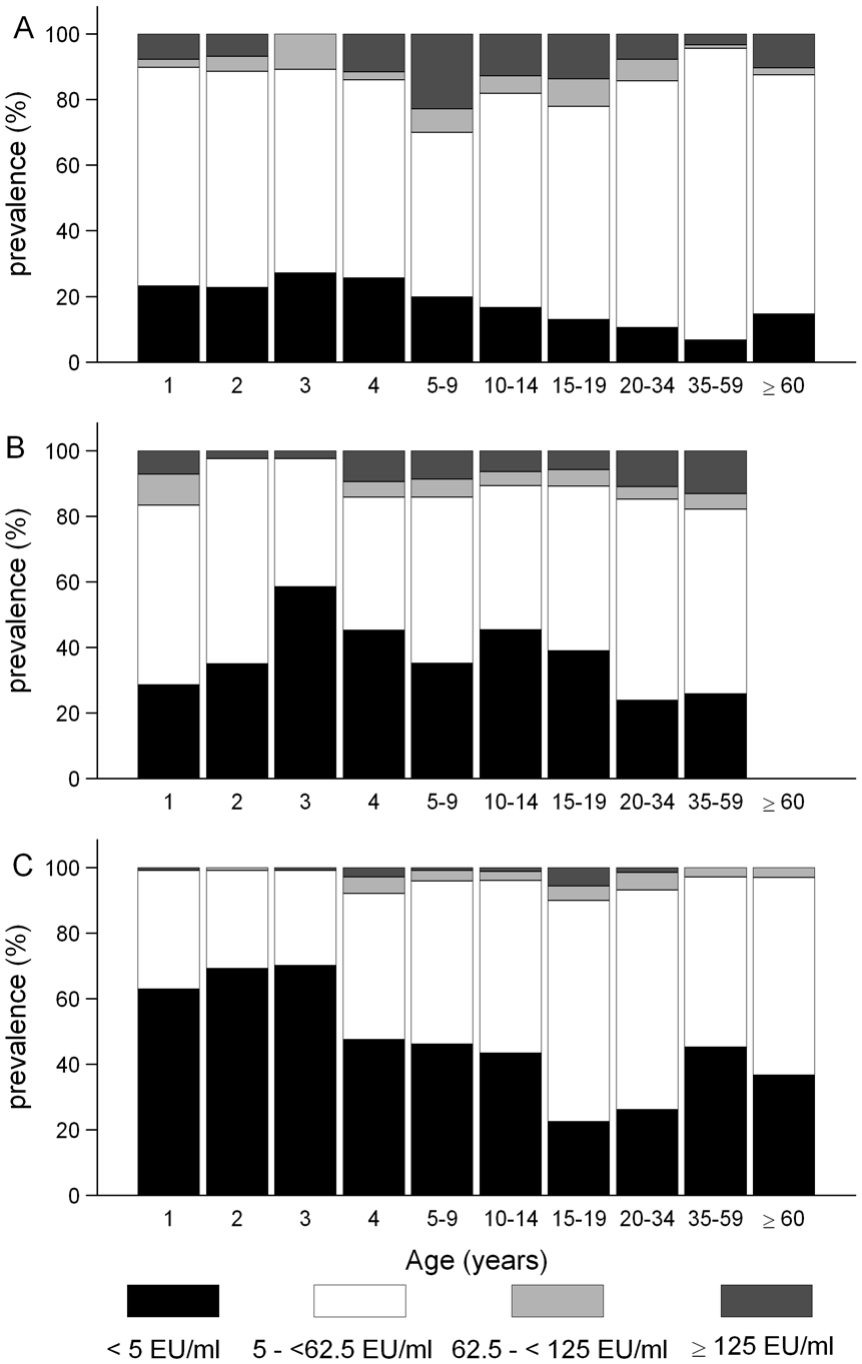
Cross-sectional distribution of anti-PT IgG levels. Distributions are shown by age (1–4 years) and age group (≥5 years). A. 1997/98; B. 2002; and C. 2007.

The cross-sectional distributions of IgG-PT levels by age (1–4 years) and age group (≥5 years) for each of the three serosurveys are shown in Figure 3. Age specific prevalence and Clopper-Pearson confidence intervals are included for the undetectable (<5 EU/ml) and high-level (≥62.5 EU/ml) categories in Table 1. In this table and subsequent discussion the term ‘high-level’, referring to antibody levels ≥62.5 EU/ml, includes those ≥125 EU/ml. Across the three surveys, there were statistically significant reductions in the population percentage with antibody levels ≥62.5 EU/ml from 19% (95% CI 16–22%) in 1997/98 to 12% (95% CI 11–14%) in 2002 and 5% (95% CI 4–6%) in 2007. Of the 19% ≥62.5 EU/ml in 1997/98, 13% (95% CI 11–16%) were ≥125 EU/ml.

**Table 1:** Australian age specific population prevalence, expressed in percentages, for Undetectable (<5 EU/ml) and High (≥62.5 EU/ml) antibody categories, with 95% Clopper-Pearson confidence intervals shown in brackets.

	1997/98			2002			2007		
Age group	*n*	<5 EU/ml	≥62.5 EU/ml	*n*	<5 EU/ml)	≥62.5 EU/ml	*n*	<5 EU/ml)	≥62.5 EU/ml
**1**	39	23 (9–43)	10 (2–27)	42	29 (14–48)	17 (6–35)	97	63 (50–74)†	1 (0–7)
**2**	44	23 (10–41)	11 (3–28)	40	35 (18–55)	3 (0–16)	97	69 (57–80)†	1 (0–7)
**3**	37	27 (12–48)	11 (3–29)	41	59 (39–76)	2 (0–16)	100	70 (58–80)	1 (0–7)
**4**	43	26 (12–45)	14 (4–31)	42	45 (27–64)	14 (4–32)	99	47 (35–60)	8 (3–17)
**1–4**	163	25 (17–34)	12 (6–19)	165	42 (33–51)	9 (5–16)	393	62 (56–68)†	3 (1–5)
**5–9**	253	20 (14–26)	30 (23–37)	231	35 (28–43)†	14 (9–21)*	493	46 (41–52)	4 (2–7)*
**10–14**	314	17 (12–22)	18 (13–24)	742	45 (41–50)†	11 (8–14)	550	43 (38–48)	4 (2–6)*
**15–19**	109	13 (6–22)	22 (13–33)	306	39 (32–46)†	11 (7–16)	317	22 (17–29)*	10 (6–15)
**20–34**	77	10 (4–22)	14 (6–26)	365	24 (19–30)	15 (11–20)	987	26 (23–30)	7 (5–9)*
**35–59**	90	7 (2–16)	4 (1–13)	190	26 (19–34)†	18 (12–25)	235	45 (37–53)†	3 (1–7)*
**60+**	48	15 (6–28)	13 (5–25)				249	37 (31–43)†	3 (1–6) *

* significantly lower than previous collection.

† significantly higher than previous collection.

The overall population percentage with undetectable antibodies increased significantly from 17% (95% CI 14–20%) in 1997/98 to 37% (95% CI 35–40%) in 2002, remaining at 38% (95% CI 36–40%) in 2007.

### Trends in antibody distribution by age

Trends in antibody distributions by age category are described, in groupings relevant to changes in the immunisation schedule over the period (Figure 1).

High-level antibody prevalence in preschool children (1- to 4-year-olds) declined markedly over the period. In infants aged from 1–2 years, this percentage fell from 17% (95% CI 6–35%) in 2002 to 1% (95% CI 0–7%) in 2007. Conversely, the percentage with undetectable antibodies increased across this entire age band from 25% (95% CI 17–34%) in 1997/98 to 42% (95% CI 33–51%) in 2002 and significantly to 62% (95% CI 56–68%) in 2007.

The 5- to 9-year-olds had the largest percentage with high-level antibody in 1997/98 with 30% (95% CI 23–37%). This significantly reduced to 14% (95% CI 9–21%) in 2002, and to 4% (95% CI 2–7%) in 2007. Undetectable antibody prevalence rose from 20% (95% CI 14–26%) in 1997/98 to 35% (95% CI 28–43%) in 2002 and 46% (95% CI 41–52%) in 2007.

Similar to observations in children aged <10 years, a reduction in the percentage of 10- to 19-year-olds with high-level antibody was observed across the first two time points, associated with a significant increase in the seronegative percentage. In contrast, however, undetectable antibody prevalence for 15- to 19-year-olds fell significantly between 2002 and 2007.

The percentage of adults (≥20 years) with undetectable antibodies increased across the surveys, from 10% (95% CI 5–15%) in 1997/98 to 25% (95% CI 20–29%) in 2002 and 31% (95% CI 28–34%) in 2007. High-level antibody prevalence fluctuated from 10% (95% CI 5–15%) in 1997/98 to 16% (95% CI 12–20%) in 2002 and 6% (95% CI 4–7%) in 2007.

## Discussion

This study compared three pertussis serosurveys undertaken in 1997/98, 2002 and 2007, finding the cross-sectional population distribution of IgG-PT levels in each survey was consistent with the prevailing pertussis epidemic cycle and changes to the nationally funded immunisation program. Whilst previous studies have used high IgG-PT levels to indicate recent infection or immunisation [4], [7], one of the most notable observations of this study was the temporal association between the high prevalence of undetectable antibodies in 2007 and the large, sustained epidemic which commenced in 2008. The significant increases in undetectable antibody prevalence between surveys suggest that waning of antibody levels, including among adults, may occur rapidly during periods of low *B. pertussis* circulation, when boosting opportunities are reduced.

Pertussis notifications prior to and during the serum collection provide the necessary epidemiologic context for interpretation of each of the cross-sectional serosurveys. Although notification rates include both confirmed and probable cases, we suspect that over time the proportion of cases notified on the basis of clinical evidence alone will have reduced, due to the increased testing options available for different ages and stages of disease. The 1997/98 collection occurred during a major epidemic, likely explaining the large population percentage with high-level antibody. The 2002 collection followed several years of moderate disease activity, consistent with the smaller number of individuals with serologic evidence of recent infection [11]. A further reduction in this number in 2007 is concordant with a relative trough in reported cases in all age groups from 2005 to 2007 [22].

The extremely low prevalence of high and very high antibody levels in the 2007 survey suggests vaccination has little impact on the presence of high level antibody at the population scale, supporting previous conclusions that high-level antibodies in 2002 were predominantly due to natural infection [7]. A pre-school booster targeting 4- to 5-year-olds has been in place since 1994, with an adolescent booster for 15- to 17-year-olds added to the NIP in 2003. Consistent with this schedule, 4-year-olds and 15- to 19-year-olds had the largest percentages of high level antibody in 2007, however, this was only around 10%, despite high estimated coverage of 89% for the 4-year-old booster [8], and moderate estimated coverage in the range 55–76% for the adolescent booster [6]. There was, however, a reduction in the percentage of 4-year-olds with undetectable antibodies compared with 3-year-olds, and similarly 15- to 19-year-olds compared with 10- to 14-year-olds in the 2007 survey. At the population level the vaccination signature may be more apparent through a reduction in the prevalence of undetectable antibody levels than an increase in the prevalence of high levels.

One of the most notable findings of this study is the increase in prevalence of undetectable antibodies in children aged from 1 to 3 years across the three collections, particularly between 2002 and 2007. High level antibodies in these ages are almost completely absent in 2007, consistent with an increased time since last vaccination compared with the same ages in 2002. These results are compatible with rapid waning of antibodies following primary vaccination with acellular vaccines [23], [24] and the withdrawal of the 18 month booster from the NIP in 2003. The temporal association with the substantial increase in notification rates which occurred in the 0–4 years age group from 2008 onwards suggests that the absence of detectable antibodies may be an indicator of increased susceptibility at the population level. An analysis of notification rates in NSW for 2008 and 2009 showed that incidence increases with age in 1–3 year olds and drops again in 4 year olds, when children receive the pre-school booster [12], further supporting increased susceptibility due to waning antibody levels.

Recent infection rates in 5- to 9-year-olds, approximated by the prevalence of high-level antibodies, were sequentially lower across the three serosurveys. This is consistent with the reducing rate of notifications in these ages, from 216 per 100,000 in 1997, to 50.6 and 19.0 per 100,000 in 2001 and 2002 respectively, and to 9.9 and 13.5 per 100,000 in 2006 and 2007 respectively [11]. These low infection rates, however, may have reduced opportunities for boosting of natural immunity and contributed to the rise in undetectable antibody levels seen in this age group. The low notification rates observed from 2001 to 2007 have not been sustained, with 115.8, 304.0, 421.1 and 544.7 cases per 100,000 in 2008 to 2011 respectively [11], again suggesting a possible link between the high percentage with undetectable antibodies in 2007 and the magnitude and duration of the subsequent epidemic. It is somewhat surprising that notification rates in this age group reached such high levels from 2009–2011, given the inclusion of a pre-school booster in the NIP since 1994. In 2007, the prevalence of undetectable antibody levels increased with age from 37% in 5 year olds to 50% in 9 year olds (data not shown), suggesting waning antibodies following the pre-school booster; notably most of this age band would have also been eligible for an 18 month booster. It is possible that the very high notification rates in this age group in 2009–2011 were mainly due to infections in 8- and 9-year olds, as occurred in previous epidemics [25]. This age cohort would not have been eligible for an 18 month booster; differential vaccine efficacy between epidemic and endemic circulation, as previously found for whole cell vaccines [26], may also be a contributory factor.

Mid-range antibody levels were predominant in the adult population in 1997/98, when infection rates were high in the under 20s and frequent natural boosting may have prevented antibodies waning to undetectable levels. Over time, however, the percentage with undetectable antibodies in adults has significantly increased. Decreasing circulation in the higher coverage acellular era may have reduced boosting opportunities, allowing waning of antibodies to non protective levels [27] and potentially creating a pool of highly susceptible adults of child bearing age. Booster doses for parents and close contacts of newborns as part of a ‘cocooning’ strategy may improve immunity in this age group [10]. In the 2007 serosurvey, the 35–59 and ≥60 years age groups had high prevalence of undetectable antibodies, and both of these age groups experienced substantially increased notification rates in the 2008–2011 epidemic, similar to other age groups with high prevalence of undetectable antibodies. It is interesting to note that the 15–19 and 20–34 years age groups, which had the lowest prevalence of undetectable antibodies in 2007, also experienced the lowest notification rates in the 2008–2011 epidemic.

With potentially fewer biases than traditional forms of surveillance, serosurveillance is increasingly being used to estimate the incidence of pertussis infection by combining information on antibody decay with seroprevalence data [16], [28]–[31]. Using a cut-off of 62.5 EU/ml to indicate infection within the previous 12 months, seroincidence in The Netherlands was estimated to be approximately 9% [29], whilst the incidence of infection in Israel was estimated at 2.4% [16]. Compared to the seroepidemiologic profiles in Finland, France and Italy collected immediately following an epidemic [15] and these results in The Netherlands and Israel, our 1997/98 results show considerably higher levels of infection. Using the same method, Australian seroincidence was around 19% in 1997/98 for the overall population (and over 30% for 5- to 9-year-olds), reducing to 12% in 2002 and 5% in 2007 (data not shown). Applying a method based on the entire antibody distribution, Kretzschmar and colleagues estimated seroincidence in five European countries ranged from 1% and 6% [30]. This method requires an assumption that endemic equilibrium has been reached, which would clearly be problematic in the Australian setting given the variation in both numbers of disease reports and seroprevalence of anti-PT antibodies observed in our survey.

Comparisons between consecutive Swedish serosurveys show similarity to our results, despite a different vaccination schedule. Overall antibody levels for both adults and children were lower in 2007 compared with 1997, ten years after the introduction of an acellular pertussis vaccination program [32]. Hallander et al. suggest that the combination of high prevalence of undetectable antibodies and low frequency of infection may indicate reduced herd immunity [24]. The distribution of IgG-PT antibodies in the Australian population in 2007 relates closely to the Swedish observations, and immediately preceded a sustained national epidemic, supporting the notion of declining herd protection.

There were some limitations to this study. Firstly, the assays for the 1997/98 survey were undertaken in a different laboratory to those in 2002 and 2007. We believe, however, that potential biases due to inter-laboratory differences were minimised through validation of the method used in all surveys against the same international standards and controls. Although the serosurveys of 2002 and 2007 showed mostly lower antibody levels than in 1997/98, we do not attribute this to inter-laboratory differences, as the levels were not consistently lower across all age groups. Furthermore, the changes in results between surveys correlated well with notification patterns which, although they may be questionable in magnitude, nevertheless reflect overall infection patterns quite well. Secondly, as the samples were drawn from specimens submitted for diagnostic testing, the vaccination status and health status of the subjects was unknown, and we cannot exclude that the sample population was not a true reflection of the general population and might have suffered from more disease. Although some seroprevalence studies have used population-based samples [29], [32], many studies have used residual sera similar to our study [14]–[16]. Convenience samples of residual sera from diagnostic testing have been shown to achieve similar results to population-based cluster sampling for the measurement of immunity to measles and mumps [17], validating our use of this method. Thirdly, unlike the Swedish study, we were not able to exclude recently vaccinated individuals [32], although our results suggest that the impact of this was minimal. There is no reason to suspect that any biases differed substantially between the three serosurvey collections.

To our knowledge, this study is the first to compare three national serosurveys conducted at different points in the pertussis epidemic cycle. Serosurvey data should be interpreted with reference to the timing of serum collection in the prevailing pertussis epidemic cycle, the historical and current pertussis immunisation program and vaccine immunogenicity. Although our data showed some evidence of vaccination, the vaccine signal was very weak and the population distribution of IgG-PT levels was dominated by natural infection and waning of antibodies in the absence of exposure or vaccination. While no defined antibody level has been identified as a single correlate of protection against pertussis, low IgG-PT levels have been shown to correlate with susceptibility to infection following household exposure [33]. Although our study design does not allow causal inferences to be made, the temporal association between increased population prevalence of undetectable antibodies and a large, sustained epidemic warrants further investigation.

The increase in the seronegative proportion between surveys supports studies which have shown antibodies wane relatively quickly after infection and vaccination, raising the possibility of a corresponding increase in the susceptible population. Further work will use mathematical models of pertussis transmission to explore the impact of vaccination changes on the population distribution of IgG-PT levels and test whether IgG-PT levels correlate with protection at the population level.
